# Monoclonal Antibodies: Historical Perspective and Current Trends in Biological Drug Development

**DOI:** 10.3390/ijms26188794

**Published:** 2025-09-10

**Authors:** Barbara Madej, Filip Tomaszewski, Dagmara Szmajda-Krygier, Rafał Świechowski, Agnieszka Jeleń, Marek Mirowski

**Affiliations:** Laboratory of Molecular Diagnostics and Pharmacogenomics, Department of Pharmaceutical Biochemistry and Molecular Diagnostics, Faculty of Pharmacy, Medical University of Lodz, Muszyńskiego 1, 90-151 Lodz, Poland; barbara.madej@stud.umed.lodz.pl (B.M.); filip.tomaszewski@stud.umed.lodz.pl (F.T.); rafal.swiechowski@umed.lodz.pl (R.Ś.); agnieszka.jelen@umed.lodz.pl (A.J.)

**Keywords:** antibody, mechanisms of action, hybridoma technology, phage display, transgenic animals, antibody drug conjugates (ADCs), bispecific antibody, nanobody

## Abstract

Antibodies, also called immunoglobulins, play a key role in the body’s immune response, binding to specific molecular targets. Of the five classes of antibodies, IgG has found the greatest clinical application. The article presents the mechanisms of antibody action, including interactions with FcR receptors on leukocytes, complement activation, and direct cytotoxic interactions, as well as the main methods of antibody production, which include hybridoma technology, phage display, and production using transgenic animals and their modifications, which allowed for the production of antibodies with reduced immunogenicity and increased their effectiveness and safety of use. It also characterizes various types of antibodies and presents the differences between them resulting from the structure and content of individual protein domains encoded by human genes and genes from other species. Antibodies are currently one of the most important groups of biological drugs used in the treatment of autoimmune, infectious, and neoplastic diseases. The properties of these large biomolecules and the achievements in the field of obtaining and modifying antibodies mean that they are currently the subject of many studies. New forms of antibodies, such as antibody–drug conjugates with highly potent cytotoxic agents, bispecific antibodies, and nanobodies, demonstrate an innovative approach to the treatment of cancer and autoimmune diseases. The dynamic development of the antibody market indicates its growing importance in modern pharmacy and medicine. Further research in this area may lead to the development of more effective and precise therapies, as well as to increase the safety of their use.

## 1. Introduction

The first attempts to use antibodies in medicine appeared at the end of the 19th century. The patients were administered antibodies from immunized animals, but the results were not very successful because serious complications occurred after their administration. Interest in antibodies was revived when, in 1975, Kohler and Milstein developed a method of obtaining monoclonal antibodies (mAbs) based on the fusion of mouse B lymphocytes with myeloma tumor cells [1,2,3]. For this discovery, both scientists received the Nobel Prize in 1984. Years of research on improving methods of obtaining antibodies, reducing side effects, and understanding the mechanisms of action and the impact of their structure on pharmacokinetic properties have resulted in almost 200 antibodies being approved for marketing in various countries around the world (according to data maintained by The Antibody Society, which provides a list of approved antibody therapeutics and those under regulatory review in the European Union and United States) [4].

This paper presents the characteristics of antibodies, their classification, mechanism of action, and methods for antibody production (hybridoma, phage display technology, transgenic animals). It also summarizes the historical development of monoclonal therapeutic antibodies, bispecific antibodies, and new directions related to nanobodies.

## 2. Basic Characteristics of Antibodies

**Figure 1:**
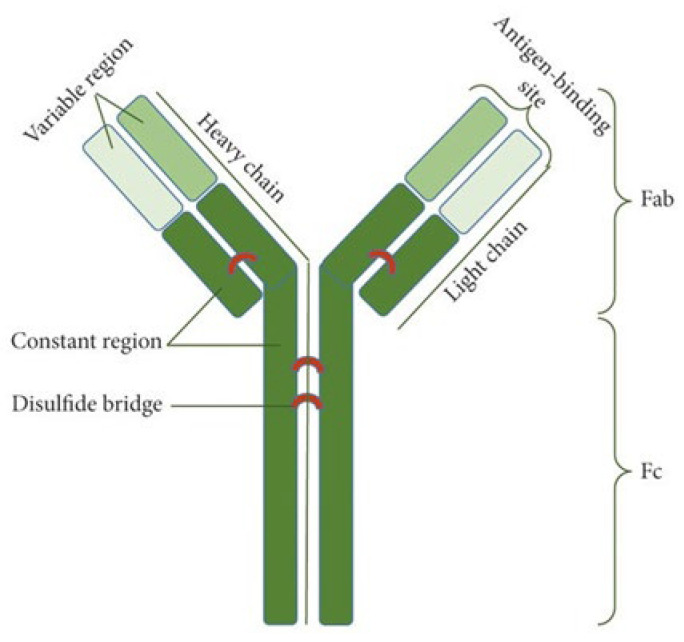
The structure of an antibody [8].

Antibodies, also called immunoglobulins, are proteins that have the ability to specifically bind to antigens. They are composed of four polypeptide chains, two light chains (LCs) and two heavy chains (HCs), which are connected by disulfide bridges. These chains contain variable parts (V) and constant parts (C). In addition, in the variable parts, hypervariable fragments are distinguished. They are responsible for the specificity of antibodies because they build antigen-binding sites. For this reason, they are also referred to as complementarity-determining regions (CDRs) [5]. The place where the antigen binds to the antibody is called the “paratope” and is composed of six hypervariable regions (three each from the light chain and the heavy chain). “Epitope”, on the other hand, refers to the surface of the antigen that binds to the antibody [6]. Studies on the interactions of CDRs with antigens have shown that the H3, H2, and L3 loops of CDRs are most frequently involved in binding to the antigen, while the L2 loop is least likely to be involved [7]. Conventional IgG antibodies are often depicted in a Y-shaped structure. Their structure includes two Fab regions (antigen-binding fragments), which are responsible for binding to the antigen and correspond to the antibody arms, and the Fc region (crystallizable fragment), with a section that binds to cellular receptors for the Fc fragment of the antibody, as well as a section that binds to complement components. This fragment is responsible for the activation of immune effector cells and the way in which abnormal or infected cells are eliminated. The Fab and Fc regions are connected to each other by a flexible hinge region [5]. The basic structure of the antibody is presented as Figure 1 according to Golpour et al. [8].

### 2.1. Classification of Antibodies

In the human body, after contact with a foreign antigen, B lymphocytes produce a range of polyclonal antibodies that bind to different epitopes on the surface of the antigen. Antibodies generated early in an immune response often show relatively low affinity for their antigen. During germinal center reactions, B lymphocytes undergo affinity maturation, which progressively increases the binding affinity of their antibodies. Avidity, by contrast, refers to the overall binding strength of multivalent antibody–antigen interactions and is distinct from affinity. Antibodies produced by different clones of B cells are called polyclonal antibodies and are found in the serum of humans and other animals [9]. Polyclonal antibodies were the first antibodies used in the treatment of various infections (e.g., hepatitis B, rabies), elimination of toxins (e.g., digoxin) from the body, or prevention of transplant rejection. In the second half of the 20th century, monoclonal antibodies began to appear, derived from a single clone of B lymphocytes [10].

There are five classes of antibodies. Due to differences in the structure of heavy chains, immunoglobulins are divided into five classes: IgG, IgM, IgA, IgD, and IgE (Table 1). Although IgM antibodies have been extensively studied, IgG remains the dominant and most clinically useful class. This group of drugs is widely used in the treatment of cancer, autoimmune diseases, and others, such as metabolic, infectious, or genetic diseases. In recent years, progress has been made in developing forms of antibodies other than monomeric IgG. IgM, due to its size, the design, expression, and purification of penta- or hexameric IgM molecules is definitely more difficult than IgG, and methods have been developed to obtain antibodies purified to a level of over 95%. About 20 IgM antibodies have been used in human trials. These molecules showed very low immunogenicity, and no serious adverse effects have been reported. However, the IgM antibodies tested so far showed a low level of efficacy, which did not allow for a positive opinion. Therefore, further research is needed to improve their clinical utility [11].

In the beginning, mouse antibodies were applied in the treatment, but they were highly immunogenic to humans. Currently, to reduce this effect, recombinant antibodies are used. They contain a small protein fraction coded by mouse gene sequences and the second major component encoded by the genes for human immunoglobulin. Depending on the percentage of these sequences in the antibody molecule, there are four types as follows:

- Mouse antibodies containing 100% mouse sequences;
- Chimeric antibodies having mouse-variable regions, which constitute about 25% of the entire sequence; the rest are human sequences;
- Humanized antibodies retain mostly human sequences, with mouse-derived CDRs and sometimes selected framework residues;
- Human antibodies, which do not contain any foreign sequences [12,13,14].

### 2.2. Mechanism of Action of Antibodies

One of the mechanisms of action of antibodies is their specific binding to Fc receptors (FcR) located on leukocytes. As a result of such binding, an immune response is triggered, leading to the destruction of the infected or cancerous cell. FcγRI binds monomeric IgG with high affinity, whereas FcγRII and FcγRIII bind IgG immune complexes with lower affinity [15]. FcγRI is characterized by high binding affinity with monomeric IgG, whereas FcγRII and FcγRIII receptors are responsible for binding polymeric IgG immune complexes to the antigen. Receptors impact the activity of the cells on which they are located (including neutrophils, monocytes, macrophages, lymphocytes) and can lead to apoptosis, phagocytosis, antibody-dependent cell-mediated cytotoxic reaction (ADCC), expression of proinflammatory cytokines, and production of hydroxyl radicals. Low-affinity receptors can only interact with multimers of immune complexes [15,16,17].

FcγRIIa and FcγRIIc are activating receptors. FcγRIIIb transmits a signal using the glycosylphosphatidylinositol domain. The remaining activating receptors have a tyrosine-based motif called the immunotyrosine-based activating motif (ITAM) [15]. Immune complexes react with the FcR group together and cause receptor aggregation. This triggers the activation of SRC family kinases (SFKs) (e.g., Lyn, Lck), which phosphorylate the ITAM domain. Spleen tyrosine kinases (SYKs) flow to the site of aggregation of immune complexes, where they become activated and lead to phosphorylation and activation of phosphoinositide 3-kinase (PI3K).

Proteins with a pleckstrin homology (PH) domain, such as Bruton tyrosine kinase (BTK), an important signaling molecule in B cells, members of VAV family proteins, which act as guanine nucleotide exchange factors (GEFs) for small G proteins of the Rho family, and phospholipase C-gamma (PLC-γ), which is activated by tyrosine phosphorylation of epidermal growth factor receptor (EGFR) and cellular homolog of the avian erythroblastosis virus oncogene B2 (c-erbB-2), are recruited. PLC-γ hydrolyzes phosphatidylinositol 4,5-bisphosphate to generate a cytosolic inositol-1,4,5 trisphosphate (IP3) and diacylglycerol (DAG). IP3 induces the release of Ca^2+^ from the endoplasmic reticulum, while DAG is responsible for activating the protein kinase C (PKC). Other signaling pathways are also activated, including a serine–tyrosine–threonine kinase (MEK), which phosphorylates and activates mitogen-activated protein kinase (MAPK) and MAP kinases, which are a family of serine/threonine protein kinases that play a crucial role in signal transduction pathways, converting extracellular stimuli into cellular responses by activation of the rat sarcoma virus (RAS) pathway. The cascade of these events leads, among others, to degranulation and release of serine proteases, leukotrienes, lysozyme, and other components from the interior of granulocytes; production of cytokines; formation of reactive oxygen species (ROS) with a strong cytotoxic effect; and oxidative burst and phagocytosis [18,19].

Among Fcγ receptors, FcγRIIb is the only inhibitory receptor. It has an immunotyrosine-based inhibitor motif (ITIM). When the immune complex binds FcγRIIb, the tyrosine of the ITIM motif is phosphorylated, but this process only occurs in the presence of clustered receptors with the ITAM motif [20]. It occurs with the participation of Lyn kinases (or another kinase from the SRC family), activated by congregated-activating receptors. The recruitment of Src homology 2 domain-containing inositol 5′-phosphatase (SHIP) and Src homology 2 domain-containing protein tyrosine phosphatase (SHP) occurs, of which SHIP-1 has the highest affinity for FcγRIIb. Phosphatases hydrolyze PtdIns (3,4,5) P3, removing one phosphate residue. In this way, the recruitment of proteins with a PH domain is inhibited, and further transmission of activating signals is interrupted.

Although FcR receptors possessing ITAMs recruit phosphatases, leading to the formation of cell activation signals, under appropriate conditions, these signals may also be inhibitory. This phenomenon has been termed ITAMi (inhibitory ITAM signaling). It is probably responsible for low-level phosphorylation of ITAM, leading to the recruitment of SYK kinases, which leads to the recruitment of SHP-1 and sends blocking signals [21].

Antibodies can also induce an immune response by binding complement components. Antigen–antibody complexes (mainly IgM and IgG) activate the classical pathway of the complement system. This occurs when the C1q molecule (a fragment of the C1 complex containing serine proteases C1r and C1s) binds to the Fc region of an antibody bound to an antigen. At this point, a cascade of reactions begins that leads to the activation of subsequent complement components. C1 autocatalysis occurs, and proteases are released, which break down C4 and C2 into smaller fragments. The individual fragments combine with each other. One of the newly formed complexes—C4bC2a (called C3 convertase)—divides the C3 complex into anaphylatoxin C3a and opsonin C3b. Opsonin covalently binds proteins and sugars on the surface of microorganisms, marking them for other cells of the immune system [22]. It can also bind to C4bC2b to form C5 convertase, which degrades C5 to C5a (another anaphylatoxin) and C5b. The C5b complex combines with C6, C7, C8, and C9 to form a membrane-activating complex (MAC), which initiates lysis of the marked cell. Thus, complement-dependent cytotoxicity (CDC) occurs via opsonization of pathogen cells, chemotaxis of C3a and C5a on leukocytes and microglial cells, and lytic cell death mediated by MAC [23].

## 3. The Methods of Producing Antibodies

### 3.1. Hybridoma Technology

**Figure 2:**
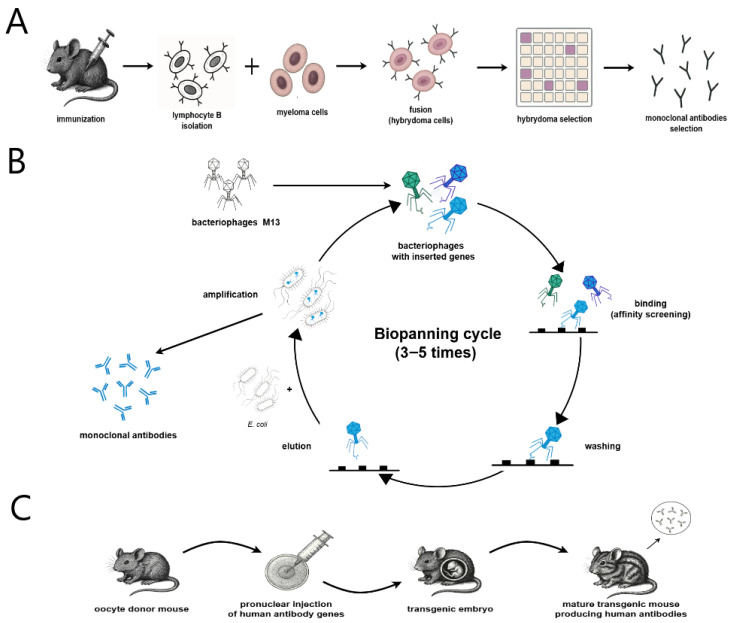
Comparison between the three methods of producing the monoclonal antibodies: (**A**) hybridoma technology; (**B**) phage display technology; (**C**) production of mAb by transgenic animals.

The method developed by Kohler and Milstein was based on the fusion of a B lymphocyte, which produces antibodies, with a cancer cell, which imparts “immortality” to the hybrid—the ability to proliferate indefinitely. In the first stage, the animal (usually a mouse) was immunized by administering the antigen against which the antibody was to be obtained (Figure 2A). Then, mature B lymphocytes producing the desired antibodies were isolated from the animal’s spleen and fused with myeloma tumor cells. Spontaneous fusion occurs very rarely, so scientists used the *Sendai virus* to facilitate it. As a result of the fusion, a new cell called a “hybridoma” was created. The fusion stage was followed by the hybridoma selection stage. Its effectiveness was influenced by many factors, including the selection of appropriate cell lines at the fusion stage or the composition of the medium in which cells are grown after fusion. Good candidates for fusion were myeloma cell lines with metabolic defects, e.g., the damaged hypoxanthine–guanine phosphoribosyltransferase (*HGPRT*) gene. An additional feature in favor of this line was the loss of the ability to produce its own immunoglobulins, which limited the time and costs associated with the isolation and purification of the desired antibodies. The enzyme encoded by the *HGPRT* gene is responsible for the synthesis of purines, so its absence means the inability to synthesize nucleotides. To prevent purine synthesis via other alternative pathways, aminopterin was added to the medium. Only hybridoma cells that had incorporated the *HGPRT* gene survived. After a few days of culture, the culture fluid should be diluted several times to obtain one hybridoma in one well. After another few days, the fluid in the wells should be examined for the presence of the desired antibodies. The received antibodies derived from one hybridoma are monoclonal antibodies [24].

The *Sendai virus* fusion method was first demonstrated in 1960, and until 1975, biological methods using viruses were the main method to facilitate cell fusion. Another virus used for this purpose was *Vesicular stomatitis Indiana virus* (VSIV)

However, these methods were superseded by the use of polyethylene glycol (PEG), first introduced in 1975. By causing cell membranes to stick together, PEG facilitated the fusion process, but the exact mechanism of its action was not known [25]. The most used was glycol, with a molecular weight of 600–6000 in 50% solutions. Fusion using PEG was performed at 37 °C for 1–3 min. PEG solutions of lower concentrations were also used, which required an extended fusion time [26]. In 1982, an improved method using PEG was presented. Lymphocyte cells were forced into close proximity with myeloma cells. These cells were supposed to approach through contact of the lymphocyte with an antigen against which the antibodies produced by the lymphocyte, or more precisely, by the BCR receptor of this antigen. The antigen was chemically bound to proteins found on myeloma cells, thus bringing both cells closer together. This procedure significantly increased the efficiency of fusion [27].

Another new method—electrofusion—appeared in 1982. Electrical pulses were used to perform fusion. They caused holes to appear in the cell membrane, which facilitated the formation of hybridomas. It has been proven that electrofusion gives better results than the use of PEG, mainly because it provides a lower probability of combining myeloma cells with inactivated lymphocytes, which increases the efficiency of obtaining normal hybridomas [28,29].

In 2009, an article was published that described a method of fusion of cells producing specific classes of antibodies. Lymphocytes expressing IgM or IgG antibodies were selected using magnetic particles containing antibodies directed against IgM or IgG on their surface. Only such selected lymphocytes were fused with myeloma cells. Thanks to the magnetic cell sorting system (MACS), in the IgM^+^ splenocyte hybridoma population, over 75% produced IgM antibodies, and in the IgG^+^ population, approximately 40% produced IgG antibodies, while not producing any IgM antibodies. This method allowed for obtaining antibodies of specific isotypes [30].

### 3.2. Phage Display

The presentation of the antibody, as well as other peptides or protein fragments, on the surface of the bacteriophage is achieved by fusion of the genes encoding the antibody with the gene encoding the structural protein of the phage (Figure 2B)—most often it is the minor envelope protein (G3P) or the major envelope protein (G8P) [31]. The most commonly used phage is M13, belonging to the group of filamentous phages, which infects *Escherichia coli* strains [32]. In order to obtain the intended antibodies, three important choices must be made: firstly, an appropriate antibody library and a source of B lymphocytes, secondly, a selection strategy (biopanning), and thirdly, a method of optimizing mAb features after selection. The best option for the use of antibodies in clinical practice is human B cells. Their source is most often the bone marrow or peripheral blood of patients, but they can also be obtained from animals or designed through an in silico antibody library. The antibody format must also be selected. The molecules displayed on the surface of the bacteriophage are usually Fab fragments or single-chain Fv fragments (scFvs), which differ in their affinity and pharmacokinetic properties. In addition to direct cloning of antibody genes into the phage genome, it is also possible to use plasmid vectors. Based on the mRNA extracted from splenocytes, complementary DNA (cDNA) is developed, which encodes a given antibody fragment. The cDNA is amplified and then cloned into a plasmid also encoding the phage coat protein gene. Such a plasmid is called a phagemid. To optimize the affinity of the antibodies, a mutation is introduced into the genes encoding the antibodies. Mutagenesis is performed by site-specific polymerase chain reaction (PCR) in complementarity-determining regions, using mutagenic *E. coli* strains or by error-prone TempliPhi DNA amplification [31]. To package the phagemid particles into *E. coli*, the helper phage M13 is used. It has a complete genome containing genes necessary for infection, replication, production, and capsid assembly. During the residence of the helper phages and phagemids in the host (*E. coli*), the wild-type G3P gene of the helper phage competes with the G3P–antibody fusion gene for incorporation into the phage. As a result, 90% of phages do not express the fusion protein genes, and among the 10% of phages that have this gene, the majority have only one copy (phages usually have three to five copies of the G3P gene). Both the phage and phagemid systems have their advantages and disadvantages. The phage system provides a wider range of antibody affinities, but phagemid systems allow for greater transformation efficiency and larger antibody libraries. The problem of low expression of fusion genes in the phagemid system was reduced by developing a method using helper phages that do not encode G3P (called hyperphages). Due to the fact that this gene is necessary for infection and packaging of the helper phage, it is necessary to obtain it from the phagemid. Therefore, phages produced in the bacterial cell must clone the fusion gene into their genome [32]. Phage display enabled the discovery of adalimumab, the first fully human antibody, later optimized for clinical use. Food and Drug Administration (FDA) approval for marketing of this drug was obtained in 2002 [12].

### 3.3. Transgenic Animals

In order to obtain human antibodies, scientists worked on various solutions—one of them was the use of transgenic mice (Figure 2C). However, introducing human genes encoding Ig into the mouse genome and obtaining the expression of these genes at a satisfactory level was not easy. In the first attempts, scientists managed to clone a 25 kb gene fragment coding the human immunoglobulin heavy chain (IgH), which was injected as a plasmid with a microinjector into fertilized mouse eggs, thus enabling its incorporation into the mouse genome. In later years, it was also possible to clone a gene fragment coding the human immunoglobulin light chain (IgL). However, the number of human antibodies in mice expressing the genes of both chains did not exceed 10% of all Ig [12]. Soon, mouse strains were obtained that did not produce their own antibodies. This effect could be obtained, for example, by deleting the mouse JH and Cκ regions, turning off the expression of the H chain or inhibiting DNA rearrangement. These methods substantially reduced mouse immunoglobulin production, enabling dominant expression of human Ig genes. Attempts were made to cross mice with human IgH and IgL genes with mice that did not produce mouse Ig and to introduce more human genes into mouse genomes. Among other things, the high expression of human V, D, and J regions allowed for obtaining a greater diversity of the antibody repertoire. Packaging a larger amount of genetic material and introducing it into the animal’s body became possible by the use of embryonic stem cells and the development of oocyte microinjection. Genes, initially packaged in plasmids, also began to be placed on bacterial artificial chromosomes (BACs) and yeast artificial chromosomes (YACs) and were introduced into embryonic cells. This procedure allowed the size of the packaged DNA to be increased from several hundred kb to about 1 Mb [33]. HuMab-Mouse and XenoMouse were the first platforms to produce fully human antibodies. After their success, biotechnology companies decided to continue this trend. In addition to transgenic mice, transgenic rats (OmniRat), chickens (OmniChicken), and (Tc Bovine), and many others have also appeared on the market. The method of preparing the immunogen, the method of immunization to which the animals are subjected, or the type of adjuvant has an impact on the size and diversity of the antibody repertoire. Undoubtedly, the use of an adjuvant significantly enhances the animal’s immune response to a given antigen. The most popular adjuvants are water/oil emulsions and aluminum salts. This response can also be enhanced by the use of molecular adjuvants, e.g., cytokines or transcription factors [34]. The first approved human monoclonal antibody obtained using transgenic animals was panitumumab (Vectibix). It was approved by the FDA in 2006. After it, several other antibodies were successfully developed, including Ustekimumab (Stelara), Ipilimumab (Yervoy), Evolocumab (Repatha), and Cemiplimab (Libtayo) [12].

## 4. Development of Therapeutic Antibodies

The antibody market is a dynamically developing branch of pharmacy and medicine. In less than 40 years until the beginning of 2025, 178 antibodies have been registered worldwide, of which 136 have been registered in the European Union. Data on therapeutic monoclonal antibodies approved by the FDA or the European Medicines Agency (EMA), such as target, year of approval, and indication for use, are available on the website www.antibodysociety.org/resources/approved-antibodies (accessed on 16 May 2025) [4].

The first monoclonal antibody approved by the FDA in 1986 was Muromonab-CD3 (Orthoclone Okt3). Muromonab bound CD3 and caused rapid depletion of T lymphocytes. Muromonab was initially approved for the treatment of acute kidney transplant rejection. Later, its indications were expanded to include the prevention of transplant rejection not only of the kidneys but also of the liver and heart [35].

Muromonab was widely used until drugs with similar but safer profiles were developed. The manufacturer decided to withdraw the drug from the market in 2010 due to the occurrence of frequent bothersome or severe side effects. Since muromonab was a mouse antibody, after introduction into the human body, it triggered a strong immune response, called the human anti-mouse antibody (HAMA) reaction, which resulted in the production of inactivating antibodies. Additionally, it often caused cytokine release syndrome that typically occurred soon after administration and could persist for hours to days. Symptoms associated with this syndrome included high fever, weakness, and chills in a large group of patients (>50%), shortness of breath, chest pain, gastrointestinal issues, such as nausea, vomiting, and diarrhea, and, less frequently, tachycardia, itching, or a rash. Less common but serious side effects occurring during muromonab treatment included aseptic meningitis, pulmonary edema, and thrombotic incidents [35,36].

The development of genetic engineering has made it possible to obtain antibodies containing fewer and fewer cross-species genes, from chimeric antibodies through humanized antibodies to fully human antibodies [37]. Thanks to these modifications, the effectiveness of antibodies was improved. Their immunogenicity, the impact of the HAMA reaction, and neutralization of antibodies were reduced, and as a result, side effects were decreased [38]. The first chimeric antibody was approved by the FDA in 1994. It was abciximab (Reopro)—an antibody directed against the GPIIb/IIIa receptor, inhibiting aggregation and preventing the formation of clots. In 1997, the first humanized antibody was approved—daclizumab (Zenapax)—an interleukin 2 (IL-2) receptor antagonist used to prevent rejection in kidney transplantation. In 2002, the first human antibody, adalimumab (Humira), received FDA approval. Adalimumab blocks tumor necrosis factor-alpha (TNF-α), and it is used, among others, in rheumatoid arthritis, psoriatic arthritis, or Crohn’s disease.

Recent research has largely focused on immunotherapy, which is based on stimulating and strengthening the immune system’s response to cancer cells. Immunotherapy can be divided into specific immunotherapy, which targets a specific type of cell to be destroyed, and nonspecific immunotherapy, which activates various immune system response mechanisms. Monoclonal antibodies can be directed against specific tumor antigens or against molecules that modulate the immune response, causing them to intensify. Additionally, monoclonal antibodies can also inhibit tumor development by blocking factors essential for their growth [39,40]. The mechanisms determining the antitumor activity of antibodies recognizing tumor antigens include direct and indirect mechanisms. Direct mechanisms primarily involve the activation of apoptosis or inhibition of signal transduction pathways by binding to membrane receptors on the surface of tumor cells. Indirect mechanisms include ADCC and CDC. There is also a group of antibodies that target specific suppressor proteins, which constitute negative checkpoints on the surface of immune cells or eliminate regulatory proteins present in the tumor microenvironment [41]. In the search for targets for anticancer immunotherapy, attention is paid to negative checkpoints, which constitute one of the mechanisms by which cells escape from the surveillance of the immune system. The best-known inhibitory immune checkpoints are programmed death receptor-1 (PD-1) and programmed death ligand 1 (PD-L1). The monoclonal antibodies currently approved for the treatment of selected cancers based on interaction of PD-1 include Niwolumab, used in melanoma immunotherapy, renal cell carcinoma (RCC), non-small cell lung carcinoma (NSCLC), head and neck squamous cell carcinoma (HNSCC), metastatic urothelial carcinoma (mUC), classical Hodgkin lymphoma (cHL), metastatic Microsatellite Instability-High (MSI-H), deficient mismatch repair (dMMR), colorectal cancer (CRC), and liver cancer. Pembrolizumab is used in cancers with dMMR, urothelial carcinoma (UCC), HNSCC, and NSCLC. Antibodies approved for the treatment of selected cancers based on interaction of PD-L1 include Atezolizumab, used in RCC and NSCLC, Durwalumab, used in mUC and NSCLC., and Awelumab for the treatment of Merkel-cell carcinoma (MCC) and mUC [42].

Furthermore, it was shown that in human epidermal growth factor receptor 2 (HER2)-enriched tumors, exposure to trastuzumab increases PD-1 and PD-L1 expression [43,44]. This increase in PD-L1 expression may be the mechanism of trastuzumab resistance [44]. An anti-PD-1 antibody substantially improved the therapeutic effect of trastuzumab [45,46]. FDA granted accelerated approval to pembrolizumab for HER2-positive gastric cancer [47,48] and nivolumab as an anti-PD-1 agent, which enhanced cytotoxic activity against A549 cells [49].

To reduce the toxicity of administered drugs and improve the effectiveness of anticancer treatment, new forms of antibodies have been developed. One of them was conjugated with highly potent cytotoxic agents or radioisotopes connected by a linker—ADC [50]. After combining with a specific target on the surface of cancer cells, antibodies reach the interior of the abnormal cell by endocytosis, where toxic substances are released in lysosomes. Since 2000, when the FDA approved Mylotarg (the first ADC), only 12 more conjugated antibodies have received FDA approval. In the European Union, 10 ADCs were registered (Table 2). Thousands of studies have been conducted to better understand their structure and the factors influencing their action. The antigen against which the antibody is directed should be selected so that it has a sufficiently high density on abnormal cells (minimum 10,000 copies per cell), which would enable endocytosis and a low density on normal cells. The next step is the selection of the appropriate linker, which can be cleavable or non-cleavable. Cleavable linkers can promote extracellular release of the substance due to the action of substances found in the blood, which can result in greater toxicity but can also increase the efficacy of action on neighboring tumor cells that express fewer antigens on the cells. Non-cleavable linkers, on the other hand, are lysed only in lysosomes as a result of the action of lysosomal enzymes, which reduces systemic side effects but also limits the effect of the substance on neighboring cancer cells. Drugs used in ADC include microtubule inhibitors, DNA-damaging agents, and topoisomerase inhibitors. These are highly toxic substances used in small concentrations (sub-nanomole) [51].

Bispecific antibodies (bsAb) represent another form of novel drugs. Their idea was to create an antibody in which each arm binds to a different epitope. One of the mechanisms of action of bispecific antibodies is to bring abnormal cells closer to cytotoxic effector cells in order to facilitate their elimination by binding one arm of the antibody to the CD3 domain of the T cell receptor and the other to the antigen on the target cell, e.g., a cancer cell. The second mechanism involves blocking signaling in abnormal cells by binding to two different molecular targets of specific signaling pathways [41,52]. Due to the structure of bispecific antibodies, they can be divided into those composed of antibody fragments, such as scFv, Fab, or single-domain antibody (sdAb) fragments, and symmetric or asymmetric antibodies based on an Fc fragment with additional Fv regions. Fragment-based bsAbs are small in size, which makes their purification easier and their penetration into tissues faster. However, their small size is also caused by faster removal from the body by the kidneys, which forces modifications to reduce the rate of elimination by combining with albumin, PEG, or other molecules. Optimizing bsAb production technology is quite a challenge. These small, powerful molecules require the development of methods to improve the stability of the molecules, reduce toxicity, and, above all, equilibrate the affinities of the bsAb arms [53]. Nowadays, 14 bsAbs are registered by the FDA and 11 are registered by the EMA (Table 3).

## 5. New Perspectives for Miniature Antibodies—Nanobodies (Nbs)

**Figure 3:**
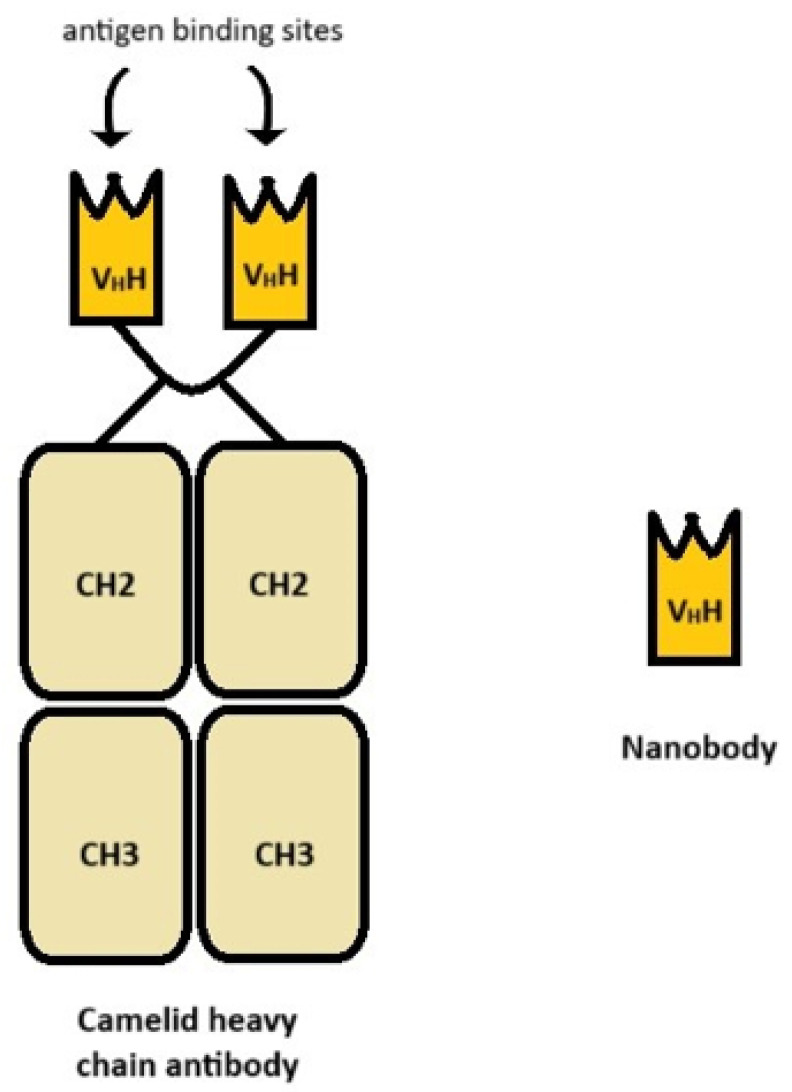
Structure of a camelid heavy chain antibody and a nanobody.

NANOBODY^®^ (registered trademark of Ablynx NV) is a fragment of IgG antibodies composed only of heavy chains, found in the *Camelidae* family. Nanobodies (Nbs) possess many unique traits in size, physical–chemical properties, immunogenicity, engineering, multiple-specific antibody construction, and more. These favorable characteristics set them apart from conventional antibodies. Their structure is presented in Figure 3. Nbs are relatively small and exhibit a broad range of binding to antigens. Unlike conventional antibodies with six CDRs per Fab, nanobodies contain a single VHH domain with three CDRs. Research has shown that this is due to the variability of the structure of the CDR1 region and the extended CDR3 region, between which there is often a disulfide bond. Another feature that distinguishes Nbs from traditional antibodies is their convex shape, which allows them to bind to antigens present on the concave part of the cell surface, inaccessible to traditional antibodies. Nbs are highly stable at high temperatures for a long time and dissolve well due to the presence of hydrophilic amino acid residues in the framework regions. Nbs are also resistant to proteases and pH changes, so they can be administered PO. They also demonstrate high tissue penetration and are even able to cross the blood–brain barrier. They are, therefore, good candidates for creating conjugates with drugs or markers, which allows for more efficient drug delivery or more accurate diagnostics. The lack of immunogenicity or very low immunogenicity also favors their use. Their small size (15kDa), although on the one hand is an advantage, on the other hand is a disadvantage because Nbs are quickly removed from the body by the kidneys, and to prevent this, the molecules must be modified. Nanobodies may have limitations in recognizing certain epitope geometries. Additionally, their production requires camelids, which are less accessible than rodent models [54,55].

More than three decades have passed since the discovery of Nbs, but their promise is growing. The numerous advantages of Nbs over conventional monoclonal antibodies and their fragments have opened up many alternative methods and techniques for generating and producing Nbs, depending on their application. Nbs are currently being used in new diagnostic tool formats, such as a lateral flow immunoassay (LFIA), diagnostic enzyme-linked immunosorbent assays (ELISAs), biosensors, and in vivo diagnostic imaging. Nb-based assays have not only found applications in disease detection but can also detect foodborne pathogens or environmental toxins. In recent years, a number of therapeutic Nbs have been approved for the treatment of cancer and autoimmune diseases. Significant potential for Nb therapy in infectious diseases has also been demonstrated. In recent years, Nbs have begun to play an important role in the development of next-generation diagnostic tools and therapies [54].

Caplacizumab (Cablivi) is the first antibody of this type, approved in 2018 by the EMA and a year later by the FDA for the treatment of Acquired Thrombotic Thrombocytopenic Purpura [56,57,58].

Ciltacabtagene autoleucel is another approved drug used in refractory/relapsed multiple myeloma recognizing B cell maturation antigen [59,60,61]. The next approved drug in Japan is Ozoralizumab, which is used in rheumatoid arthritis targeting tumor necrosis factor-alpha [62]. A large group of nanobodies is being widely tested, and some of them are in various phases of clinical trials. In phase 3, Gefurulimab was tested against myasthenia gravis, which aimed autoantibodies against acetylcholine receptors [63]. In phase 2, 68-GaNOTA-Anti-HER2 VHH11 was tested against HER2-positive breast carcinoma [64,65]. Vobarilizumab (ALX-0061) was intended to eliminate symptoms of rheumatoid arthritis and systemic lupus erythematosus by blocking the interleukin-6 receptor [66,67]. Sonelokimab (M1095) was used against Psoriasis by targeting interleukin-17A/F [68]. NbV565 was used against Crohn’s disease and was aimed at tumor necrosis factor [69]. ARP1 and VHH batch 203027 were used to prevent diarrhea caused by rotavirus [70]. ALX-0171 was intended for protection against lower respiratory tract infection caused by respiratory syncytial virus [71]. LMN-101 was used to eliminate the infection caused by Campylo bacteriosis, recognizing Campylo bacterjejuni [72]. In the first phase of clinical trials, there is isotopically labeled Nb 131I-GMIB-anti-HER2 VHH1 targeting the HER2 receptor, which is overexpressed in breast carcinoma [73], and M6495, which is used against osteoarthritis, hitting disintegrin and metalloproteinase with thrombospondin motif-5 [74].

The use of nanobodies in cancer therapy also seems very promising. Using chimeric antigen receptor (CAR) T cells collected from patients that are genetically modified in vitro to express a nanobody specific for the tumor antigen allows the T cells, when re-administered to the patient, to recognize tumor cells and eliminate them by releasing cytotoxic molecules, inducing apoptosis through recognition of the tumor necrosis factor receptor, and secreting inflammatory cytokines [75].

Another emerging perspective for the use of nanobodies in nanomedicine and nano-oncology is their conjugation with nanoparticles. Such conjugates can be targeted to diseased tissues, and the nanoparticle can contain a drug that can be selectively delivered there. Compared to monoclonal antibodies, nanobodies are characterized by their smaller size, which is interesting for the development of new therapeutic strategies. They may be better at targeting antigens found in poorly vascularized and difficult-to-access tissues. Nanobodies demonstrate better tissue extravasation and penetration than classic monoclonal antibodies, which is obviously crucial for therapeutic applications [76,77]. Thus, many nanobodies have been generated to inhibit vascularization [78]. A nanobody directed against VEGFR2 has also been identified, with high antigen-binding affinity (KD = 5.4 nM) and the potential to inhibit capillary formation in vitro [78]. To date, nanobodies have been primarily used in therapies targeting extracellular targets, such as ligand receptors and transmembrane proteins differentially expressed in cancer target cells. Nanobodies have been developed against the transmembrane growth factor receptors EGFR1 (HER1), EGFR2 (HER2), VEGFR2, c-Met, and chemokine receptor type 7 (CXCR7) [77,79]. Overexpression of these receptors typically accompanies malignancies. Significant VEGFR expression has been reported in brain, lung, breast, and colon cancers; c-Met is associated with colon, breast, and ovarian cancers, as well as hematological malignancies; and overexpression of CXCR7 correlates with breast and lung cancer [77,80]. Nanobodies have also been developed against extracellular targets, such as hepatocyte growth factor (HGF) and chemokines [81]. Nanobodies can be used as drug carriers (nanocarriers) and other delivery systems in targeted therapy. These conjugates reduce systemic toxicity, improve drug solubility in lipid bilayers or micelles, and allow for higher single-dose administration [82]. Examples of such systems include nanobodies–liposomes [83], nanobodies–micelles [84], and nanobody–albumin nanoparticles [85] with increased specificity for EGFR-expressing cancer cells. Nanobodies have also been attempted as antagonists to prevent ligand binding and induce conformational changes that lead to the activation of signaling cascades or modulate the enzymatic activity of target proteins [77]. It should be emphasized that anti-ligand nanoparticles can only be effective when a single ligand is involved in the induction of the receptor signaling cascade [82].

Nanobodies have also been attempted as antagonists to prevent ligand binding and induce conformational changes that lead to the activation of signaling cascades or modulate the enzymatic activity of target proteins [77]. It should be emphasized that anti-ligand nanoparticles can only be effective when a single ligand is involved in the induction of the receptor signaling cascade [82]. Nanobodies demonstrate good stability in harsh environments, so administration can be intravenous, oral, intraperitoneal, or intratumoral. Monomeric nanobodies are rapidly cleared from the bloodstream via the kidneys, and if such nanobodies are conjugated with toxic substances, their accumulation in the kidneys can lead to undesirable renal toxicity [65]. Nanobodies were conjugated with various nanoparticles [82], e.g., liposomes. By targeting EGFR overexpressed on cancer cells, Oliveira et al. [83] demonstrated better binding of nanobody-conjugated liposomes to human cancer cells compared to liposomes without a conjugated nanobody. These systems were improved [86], and new polymeric micelles conjugated with anti-EGFR nanobodies (EGa1) [84,87] and albumin-conjugated nanoparticles (anti-EGFR nanobody–albumin nanobody) [85] were developed. The above data indicate the potential of nanobodies for their future use in targeted therapies. Nanobodies that have entered clinical trials are summarized in the article by Jovčevska and Muyldermans [88].

## 6. Personal Opinion

In our opinion, antibodies continue to hold a great promise for the development of new therapies for autoimmune, infectious, and neoplastic diseases. We consider the methods for antibody production, including hybridoma technology, phage display, and production using transgenic animals, as well as their various modifications, to be remarkable achievements. During the production process, many of their disadvantages, such as immunogenicity, appear to have been reduced, which has increased their efficacy and safety. From our perspective, antibodies currently constitute one of the most important groups of biological therapeutics. We are particularly excited about new forms of antibodies, such as antibody–drug conjugates with potent cytostatics, bispecific antibodies, and nanobodies conjugated with nanoparticles, as they seem to offer promising prospects for use in nanomedicine and nano-oncology. We hope their novel forms of application will further revolutionize the use of these molecules in pharmacotherapy, particularly in terms of improving safety, efficacy, and accessibility, which may contribute to reducing the costs of such drugs.

## 7. Conclusions

Monoclonal antibodies have revolutionized the treatment of many diseases, especially cancers (e.g., leukemia, lymphoma, breast cancer) and autoimmune diseases (e.g., lupus, psoriasis, multiple sclerosis, ulcerative colitis). Antibodies act selectively on specific molecular targets and enable targeted therapy. They are characterized by significantly lower toxicity compared to classical chemotherapy. Compared with chemotherapy, antibodies generally exhibit reduced systemic toxicity, though immune-related adverse events can still occur. However, they are not devoid of causing adverse effects, such as nausea, vomiting, immunosuppression, and the associated neutropenia and opportunistic infections, as well as thrombocytopenia. Nevertheless, thanks to therapy monitoring, treatment safety increases, and the effectiveness in many difficult cases is significant. Current research focuses on the development of bispecific antibodies (combining two different epitopes), combination therapies (with cytotoxic drugs, radioisotopes), and the development of fully human antibodies and antibody fragments (e.g., nanobodies) with smaller size and improved tissue penetration. Antibody fragments are being investigated for alternative administration routes, such as oral or inhalation delivery.

## Figures and Tables

**Table 1:** Characteristics of antibody classes.

Class	Form	Half-Life (Days)	Serum Concentration (mg/mL)	Percentage in Serum	Description
IgG	monomer	23	8–16	80	They play the most important role in the immune response in defending against microorganisms and viruses that have penetrated cells and cancer cells, initiating the death of these cells.
IgA	monomer, dimer, tetramer	5.8	1.4–4	13	They perform protective functions against pathogens in the mucous and serous membranes; they are found in tears, sweat, and secretions from the glands of the digestive, respiratory, and urinary tracts.
IgM	pentamer, hexamer	5.1	0.5–2	6	They are produced first in the initial phase of the immune response; their affinity for the antigen is low.
IgD	monomer	2.8	0.04	0.001	They are abundant on the surface of B lymphocytes (not in contact with the antigen) as their receptors; they are scarce in the serum; when bound to basophils and mast cells, they inhibit their degranulation and allergic reactions.
IgE	monomer	2.5	0.00002–0.0005	0.000003	They participate in defense against parasites and allergies; by binding to basophils and mast cells, they cause their degranulation, releasing, among others, histamine.

**Table 2:** Antibody–drug conjugates approved in the EU.

INN (Brand Name)	Target	Drug	Indication	Approval Year
Ibritumomab tiuxetan (Zevalin) ^1^	CD20	[90Y]	Follicular lymphoma	2004
Brentuximab vedotin (Adcetris)	CD30	MMAE	Hodgkin’s lymphoma, systemic anaplastic large cell lymphoma, cutaneous T cell lymphoma	2012
Trastuzumab emtansine (Kadcyla)	HER-2	DM1	Breast cancer	2013
Gemtuzumab ozogamicin (Mylotarg)	CD33	N-acetyl-gamma-calicheamicin	Acute myeloid leukemia	2018
Polatuzumab vedotin (Polivy)	CD79b	MMAE	Diffuse large B cell lymphoma	2020
Belantamab mafodotin (BLENREP) ^1^	BCMA	mcMMAF	Multiple myeloma	2020
Trastuzumab deruxtecan (Enhertu)	HER-2	deruxtecan	Breast cancer, non-small cell lung cancer, stomach cancer	2021
Sacituzumab govitecan (Trodelvy)	TROP-2	SN-38	Breast cancer	2021
Enfortumab vedotin (Padcev)	Nectin-4	MMAE	Urothelial cancer	2022
Loncastuximab tesirine (Zynlonta)	CD19	SG3249 (tesirine)	Diffuse large B cell lymphoma, high-grade B cell lymphoma	2022

^1^ Withdrawal or authorization has lapsed. MMAE—monomethyl auristatin E. DM1—mertansine. mcMMAF—maleimidocaproyl monomethylauristatin F. SN-38—active metabolite of irinotecan.

**Table 3:** Bispecific antibodies approved in the EU.

INN (Brand Name)	Target	Indication	Approval Year
Catumaxomab (Removab) ^1^	EpCAM, CD3	Neoplastic ascites	2009
Blinatumomab (Blincyto)	CD19, CD3	Acute lymphoblastic leukemia	2015
Amivantamab (Rybrevant)	EGFR, MET	Non-small cell lung cancer	2021
Faricimab (Vabysmo)	Ang-2, VEGR-A	Neovascular age-related macular degeneration, visual impairment due to diabetic macular edema	2022
Mosunetuzumab (Lunsumio)	CD20, CD3	Follicular lymphoma	2022
Teclistamab (TECVAYLI)	CD3, BCMA	Multiple myeloma	2022
Glofitamab (Columvi)	CD20, CD3	Diffuse large B cell lymphoma	2023
Epcoritamab (TEPKINLY)	CD20, CD3	Diffuse large B cell lymphoma, follicular lymphoma	2023
Talquetamab (TALVEY)	Protein G (GPRC5D), CD3	Multiple myeloma	2023
Elranatamab (Elrexfio)	CD3, BCMA	Multiple myeloma	2023
Odronekstamab (Ordspono)	CD20, CD3	Diffuse large B cell lymphoma, follicular lymphoma	2024

^1^ Withdrawal or authorization has lapsed.

## Abbreviations

The following abbreviations are used in this manuscript:

ADC	antibody–drug conjugate
ADCC	antibody-dependent cell-mediated cytotoxic reaction
BAC	bacterial artificial chromosome
bsAb	bispecific antibody
BTK	Bruton tyrosine kinase
CAR	chimeric antigen receptor
CDC	complement-dependent cytotoxicity
cDNA	complementary DNA
CDR	complementarity-determining region
c-erbB-2	cellular homolog of the avian erythroblastosis virus oncogene B2
cHL	classical Hodgkin lymphoma
CRC	colorectal cancer
CXCR7	chemokine receptor type 7
DAG	diacylglycerol
dMMR	deficient mismatch repair
EGFR	epidermal growth factor receptor
ELISA	enzyme-linked immunosorbent assay
EMA	European Medicines Agency
Fab	antigen-binding fragment
Fc	crystallizable fragment
FcR	Fc receptor
FDA	Food and Drug Administration
G3P	minor envelope protein
G8P	major envelope protein
GEF	guanine nucleotide exchange factor
HAMA	human anti-mouse antibody
HC	heavy chain
HER2	human epidermal growth factor receptor 2
HGF	hepatocyte growth factor
HGPRT	hypoxanthine–guanine phosphoribosyltransferase
HNSCC	head and neck squamous cell carcinoma
IgH	immunoglobulin heavy chain
IgL	immunoglobulin light chain
IL-2	interleukin 2
IP3	cytosolic inositol-1,4,5 trisphosphate
ITAM	immunotyrosine-based activating motif
ITIM	immunotyrosine-based inhibitor motif
LC	light chain
LFIA	lateral flow immunoassay
mAb	monoclonal antibody
MAC	membrane-activating complex
MACS	magnetic cell sorting system
MAPK	mitogen-activated protein kinase
MCC	Merkel-cell carcinoma
MEK	serine–tyrosine–threonine kinase
MSI-H	microsatellite instability-high
mUC	metastatic urothelial carcinoma
Nbs	nanobody
NSCLC	non-small cell lung carcinoma
PCR	polymerase chain reaction
PD-1	programmed death receptor-1
PD-L1	programmed death ligand 1
PEG	polyethylene glycol
PH	pleckstrin homology
PI3K	phosphoinositide 3-kinase
PKC	protein kinase C
PLC-γ	phospho-lipase C-gamma
RAS	rat sarcoma virus
RCC	renal cell carcinoma
ROS	reactive oxygen species
scFv	single-chain Fv fragment
sdAb	single-domain antibody
SFK	SRC family kinase
SHIP	Src homology 2 domain-containing inositol 5′-phosphatase
SHP	Src homology 2 domain-containing protein tyrosine phosphatase
SYK	spleen tyrosine kinase
TNF-α	tumor necrosis factor-alpha
UCC	urothelial carcinoma
VEGFR2	vascular endothelial growth factor receptor 2
VSIV	vesicular stomatitis Indiana virus
YAC	yeast artificial chromosome
